# Measurements of AMPs in stratum corneum of atopic dermatitis and healthy skin–tape stripping technique

**DOI:** 10.1038/s41598-018-20204-8

**Published:** 2018-01-26

**Authors:** Maja-Lisa Clausen, H.-C. Slotved, Karen A. Krogfelt, Tove Agner

**Affiliations:** 1 Department of Dermatology, Bispebjerg University Hospital, Copenhagen, Denmark; 2 Department of Bacteria, parasites and fungi, Statens Serum Institut, Copenhagen, Denmark

## Abstract

Decreased levels of antimicrobial peptides (AMPs) in atopic dermatitis (AD) have previously been reported and have been linked to the increased susceptibility to skin infections found in AD patients. This study intents to identify AMPs: hBD-2, hBD-3, RNase7, psoriasin and LL-37 in AD patients and healthy controls, and determine concentrations in consecutive depths of the outer most skin layers. Tape stripping was used on lesional and non-lesional skin. From each skin site, 35 consecutive tape strips were collected and pooled in groups of 5. Commercially available ELISA kits were used to determine AMP concentration in stratum corneum samples. hBD-2, hBD-3, RNase7 and psoriasin were identified in stratum corneum samples. hBD-3-level was markedly higher in AD non-lesional skin compared to healthy controls, and a similar trend was observed for RNase7. Most AMPs were distributed evenly through 35 tape strips, implying a homogeneous distribution of antimicrobial defense in the outer most skin layers. The findings indicate that AD patients may not suffer from a general baseline deficiency in AMPs, and that the innate immune defense is present throughout the stratum corneum, both insights of importance for understanding the role of AMPs in AD.

## Introduction

Atopic dermatitis (AD) is a common skin disease characterized by dysfunctional immunological response, skin barrier defects and frequent skin infections. It is a chronic, relapsing skin disease, affecting up to 20% of children and up to 10% of adults worldwide^1^. A major problem in the management and treatment of AD is recurrent skin infections with *Staphylococcus aureus*^2^, resulting in repeated flares, sustained disease activity and frequent antibiotic treatment. This increased susceptibility to skin infections has been attributed to decreased levels of antimicrobial peptides (AMPs)^3^. AMPs act as direct antimicrobial effector molecules, creating an important first line of defense in the skin, and possess broad antimicrobial activity^4^. Furthermore, AMPs are essential immune mediators, linking the adaptive and innate immune response, regulating cytokine response, attracting immune cells as well as interacting with the skin microbiome^5–7^. Multifaceted functions of AMPs are described, promoting immunity as well as participating in the pathogenesis of certain inflammatory diseases^8,9^, making them interesting targets for therapeutic development and treatment of inflammatory diseases.

Until now most studies on AMPs in AD skin have identified AMPs in full thickness skin biopsies^3,10–13^ and only few studies using skin washing fluid, and tape stripping technique have been presented^14–16^. The aim of the present study was to determine the concentration of different AMPs in lesional and non-lesional AD skin, and in healthy control skin, using tape stripping technique and commercially available ELISA kits, with focus on stratum corneum, where the antimicrobial function is anticipated to be of greatest importance.

## Results

### Clinical characteristics

SCORAD for AD patients ranged from 7.7–61.5, with a median of 33.8 (25–75^th^ percentile = 18.28–44.18) (Table 1). TEWL (baseline) was significantly higher in AD non-lesional skin (median TEWL = 9.2, 25–75^th^ percentile = 6.95–12.6) compared to healthy control skin (median TEWL = 5.9, 25–75^th^ percentile = 5.2–6.85) p = 0.04. TEWL after tape stripping was measured, and resulted in a median increase in TEWL of 60.95 g/cm2/h (25–75^th^ percentile = 47.08–76.83). All participants showed a noticeably increase in TEWL after tape stripping, apart from two patients. After tape stripping, median TEWL for AD patients was 63.4 g/cm^2^/h (25–75^th^ percentile = 37.8–77.2), and median TEWL for healthy controls was 80.9 g/cm^2^/h (25–75^th^ percentile = 67.75–92.4) (Table 1).

**Table 1:** AMP concentration and clinical parameters.

	Skin	AD1	AD2	AD3	AD4	AD5	AD6	AD7	AD8	AD9	HC1	HC2	HC3	HC4	HC5
hBD-2 *pg/µg protein*	nls	0	0	3.2	0	0.2	0	0.2	0	0.2	0.2	0.2	0	0.1	0
	ls	0	1.7	1.4	0.6	4.5	0.1	0	3.6	2.5					
hBD-3 *pg/µg protein*	nls	181	276.9	213.1	86.6	356.2	384.5	204.9	510.1	305	194.9	142.2	77.6	140.4	97.3
	ls	341.6	889.3	178.5	101	338.3	410.9	581.4	625.1	85.8					
RNase7 *ng/µg protein*	nls	3.7	5.6	0.6	1.8	3.2	3.1	3.7	5.3	4.7	1.8	2.2	2.5	3.1	2.1
	ls	6.1	5.3	1.7	1.7	2.3	5.9	3.7	8.7	2.1					
Psoriasin	nls	0	0	0	0	0	0	0	0	0.5	0	0	0	1.0	2.6
	ls	0	0.7	0	0.3	0.8	0.5	0.6	0.7	1.3					
TEWL baseline (nls) g/m^2^h	nls	12.1	9.2	16.8	8.5	4.6	7.7	6.2	11.4	13.1	7.2	5.5	6.5	4.9	5.9
TEWL after tape stripping (nls) g/m^2^h	nls	74.9	88.5	52.4	21.7	58.9	79.5	23.2	63.4	72.2	95.4	78.7	89.4	80.9	56.8
SCORAD	NA	7.7	43.7	61.5	45.6	39.9	36.8	30.8	21.8	30	NA	NA	NA	NA	NA
Filaggrin gene mutations	NA	wildtype	Het R501X	wildtype	wildtype	wildtype	wildtype	wildtype	Het R501X	wildtype	wildtype	wildtype	wildtype	wildtype	wildtype

Mean AMP concentration, trans epidermal water loss (TEWL), eczema severity (SCORAD) and filaggrin gene mutation status for each individual atopic dermatitis patient (AD) and healthy control (HC).

NA: not applicable; nls: non-lesional skin; ls: lesional skin.

### AMPs measured in stratum corneum from AD patients and healthy controls

**Figure 1:**
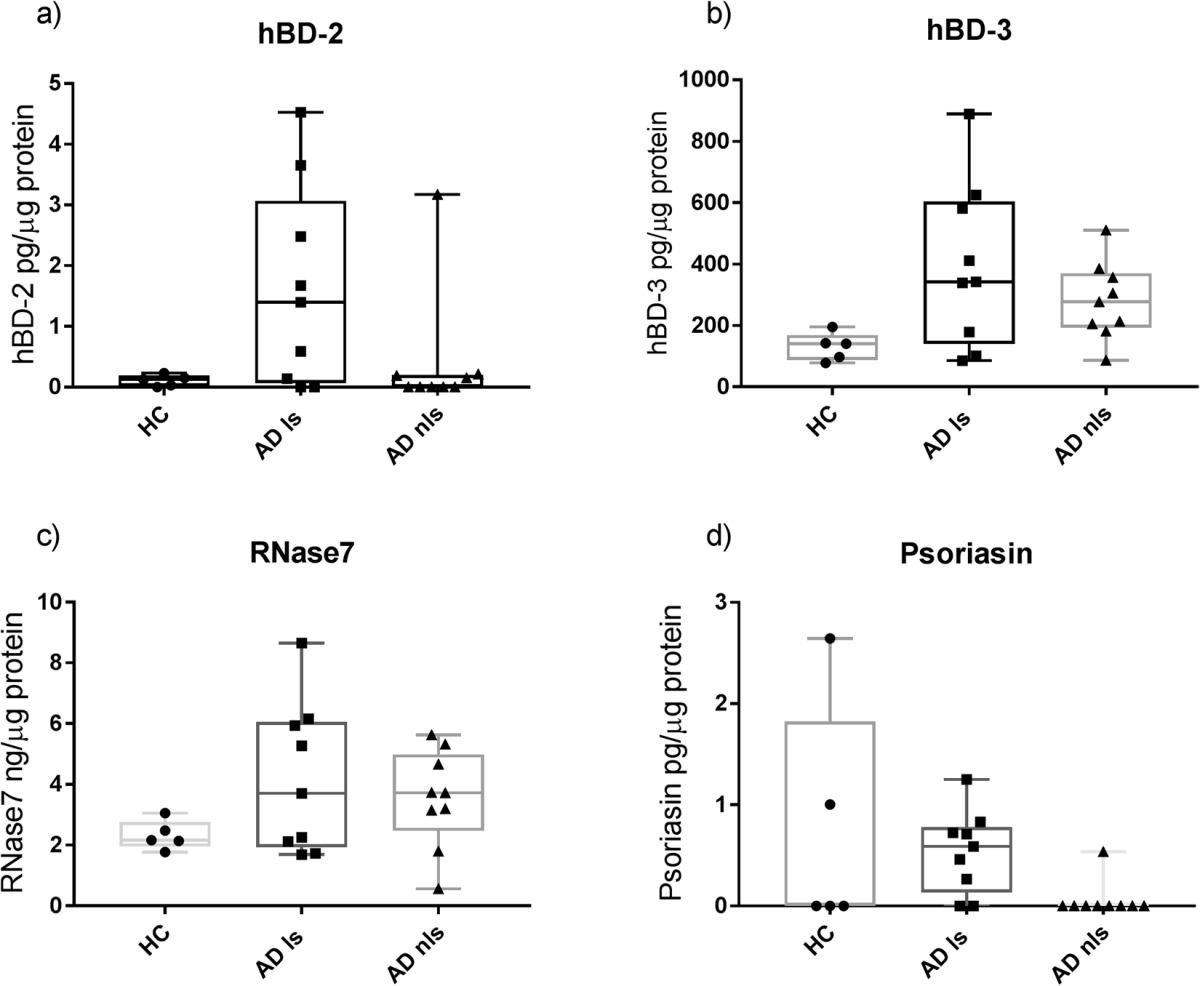
AMPs in AD skin and healthy skin. Concentration of antimicrobial peptides (AMPs) in stratum corneum in AD lesional skin (AD ls), AD non-lesional skin (AD nls) and healthy control skin (HC). AMP content is expressed per µg of total protein per sample in stratum corneum tape strip samples. The content of each AMP was determined by ELISA analysis. Results are shown as box-plots (min, 25^th^ percentile, median, 75^th^ percentile, max). AD: n = 9, HC: n = 5.

Human beta-defensin-2 (hBD-2), human beta-defensin-3 (hBD-3), Ribonuclease 7 (RNase7) and psoriasin were all identified in stratum corneum, while cathelicidin (LL-37) was not (Tables 1 and 2). Inter-individual variation with respect to AMPs was highest in AD lesional skin and lowest in healthy skin (Fig. 1). hBD-2 was detected primarily in AD lesional skin, and only in few samples of AD non-lesional skin and healthy controls (Table 2). No significant differences in the amount of hBD-2 between lesional, non-lesional and healthy skin were found. hBD-3 was detected in all skin samples from AD patients and healthy controls. hBD-3 was significantly higher in AD non-lesional skin compared to healthy skin (p = 0.019) (Table 2, Fig. 1). RNase7 was detected in all samples from AD lesional skin and non-lesional and healthy controls, and as for HBD-3 there was a trend towards higher concentrations in AD non-lesional skin compared to healthy skin (Table 2). Psoriasin was detected predominantly in AD lesional skin, and only sporadically in AD non-lesional skin and healthy controls (Table 2). For psoriasin, concentrations were below the lowest standard of the standard curve, but above the detection limit of the ELISA kit. LL-37 was not detected in any stratum corneum samples.

**Table 2:** AMP concentration in AD and healthy skin.

AMP*	AD lesional skin n = 9	AD non-lesional skin n = 9	Healthy controls n = 5	p-value (CI of difference)
hBD-2				
Patients with detected hBD-2	7	4	4	
*Concentration of hBD-2; pg/µg protein, median (25/75 quartiles)*	1.4 *(0.1–3.1)*	0 *(0–0.2)*	0.1 *(0–0.2)*	AD ls-AD nls *p = 0.15 (−3.7 to 0.2)* AD ls-HC *p = 0.15 (−0.03 to 3.5)* AD nls-HC *p = 0.61 (−0.2 to 0.2)*
hBD-3				
Patients with detected hBD-3	9	9	5	
*Concentration of hBD-3; pg/µg protein, median (25/75 quartiles)*	341.6 *(139.8–603.3)*	276.9 *(193–370.4)*	140.4 *(87.4–168.6)*	AD ls- AD nls *p = 0.30 (−376 to 34)* AD ls- HC *p = 0.06 (−11 to 503)* **AD nls-HC** ***p = 0.019**** (18 to 278)*
RNase7				
Patients with detected RNase7	9	9	5	
*Concentration of RNase7; ng/µg protein, median (25/75 quartiles)*	3.7 *(1.9–6.0)*	3.7 *(2.5–5.0)*	2.2 *(1.9–2.8)*	AD ls- AD nls *p = 0.57 (−2.8 to 0.94)* AD ls- HC *p = 0.44 (−0.5 to 4)* AD nls- HC *p = 0.08 (−0.4 to 2.9)*
Psoriasin				
Patients with detected Psoriasin	7	1	3	
*Concentration of Psoriasin; pg/µg protein, median (25/75 quartiles)*	0.6 *(0.1–0.8)*	0 (0)	0 (0–1.8)	AD ls- AD nls *p = 0.016 (−0.7 to 0)* AD ls- HC *p = 0.79 (−1.8 to 0–7)* AD nls- HC *p = 0.34 (−2.6 to 0)*
LL-37				
Patients with detected LL-37 *ng/µg protein*	Not detected	Not detected	Not detected	—

Concentration of each AMP in AD lesional skin (AD ls), AD non-lesional skin (AD nls) and healthy control skin (HC). Results are given as median values with 25–75 percentiles in brackets. For each AMP, medians are calculated from 7 samples taken from each AD patient (n = 9) comprising 63 samples and 7 samples taken from each healthy control (n = 5) comprising 35 samples.

*AMP concentration is calculated as AMP/ µg of total protein for each sample.

### AMP concentration in consecutive depths of stratum corneum

**Figure 2:**
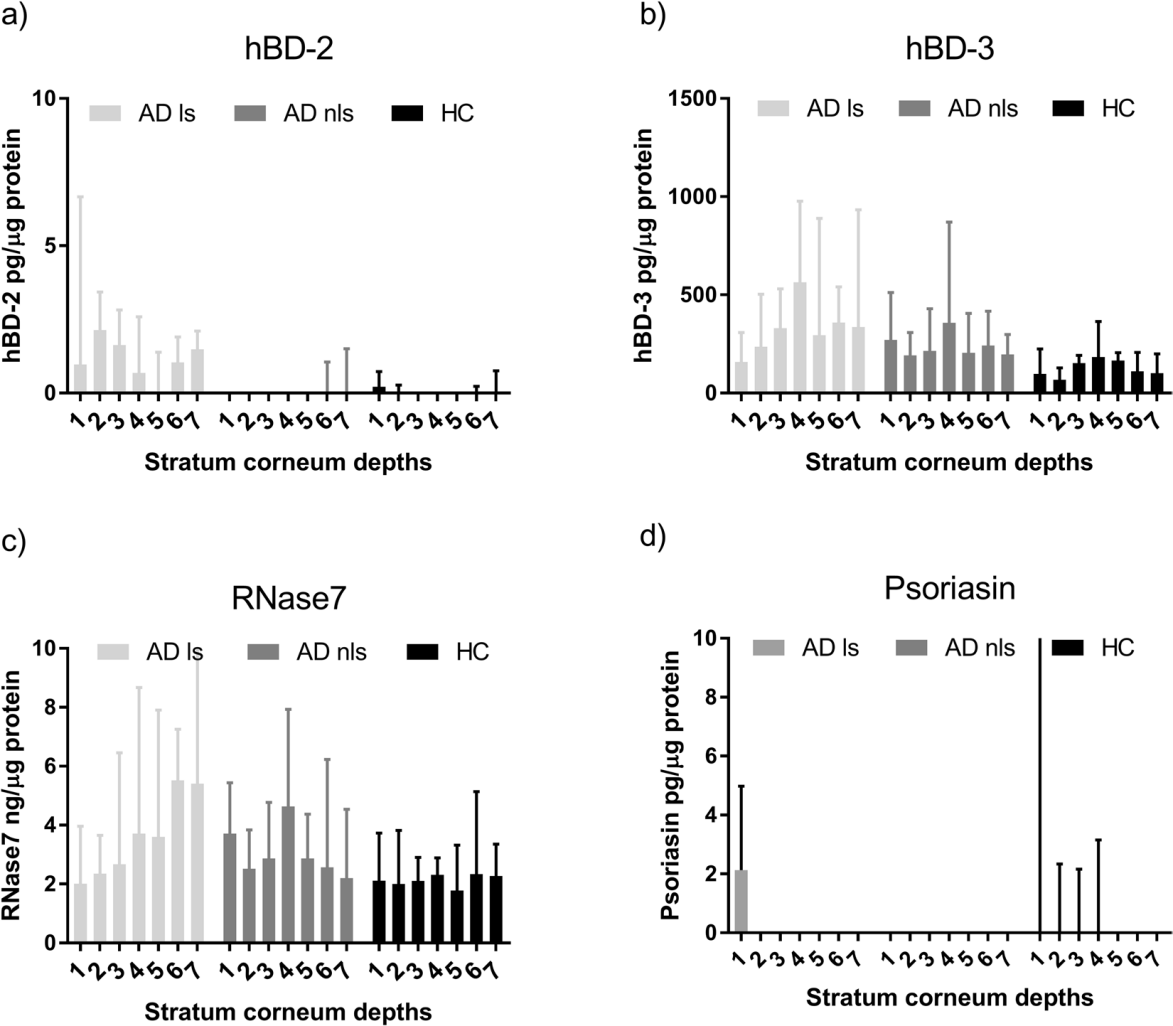
AMP concentration in different stratum corneum depths. Concentration of each antimicrobial peptide (AMP) in atopic dermatitis (AD) and healthy control (HC) skin, for every depth of stratum corneum (1–7). The AMP content is determined per µg of total protein per sample, and shown for each skin type: AD lesional skin (AD ls), AD non-lesional skin (AD nls) and healthy control skin (HC). Results are given as median values ± 95% CI of 9 AD samples and 5 HC samples for each stratum corneum depth.

AMPs were detected through 35 consecutive tape strips of stratum corneum. Concentrations of AMPs showed no obvious change with increasing depth, except for psoriasin, which was detected mainly in the outermost layers, and not in the deeper samples of stratum corneum (Fig. 2). For RNase7 in AD lesional skin there was a trend of increasing concentration with increasing depth in stratum corneum, with 2.01 ng/µg protein at depth 1 and 5.4 ng/µg protein at depth 7. In AD non-lesional skin and healthy skin a more stable concentration of RNase7 was observed (Fig. 2, Table S1).

### Validation of ELISA by spiking

All ELISAs detected the tested recombinant peptide and quality control. Despite positive spike test, optical density (OD) measurements were for some recombinant peptides lower than expected. This is most likely due to different immunogen binding sites between natural and recombinant peptides (Supplementary Fig. S2). Synthesized peptide for hBD-3 from Alpha Diagnostics was not positive on the ELISA from Cloud Clone.

## Discussion

Focusing on stratum corneum, and using a minimal invasive tape stripping technique together with commercially available ELISAs, four different AMPs (hBD-2, hBD-3, RNase7 and psoriasin) were identified in AD patients and healthy controls. hBD-3 expression was higher in AD non-lesional skin compared to healthy controls. A similar trend was observed for RNase7, indicating that baseline values of AMPs are not generally decreased in AD patients. AMPs were found to be evenly distributed in consecutive layers of the stratum corneum, demonstrating a homogeneous antimicrobial defense independent of depth.

Using tape stripping technique and commercially available ELISAs, we were able to detect AMP presence in stratum corneum, in line with previous reports using skin biopsies for mRNA and protein analyses^10,16–20^. Tape stripping for collection of material for AMP assessment ensures focus on stratum corneum, which is where AMPs are presumed to play an important role in the direct antimicrobial defense. The technique minimizes the influence of other tissues on the measurements, and has previously been used for the detection of proteins, mRNA, cytokines and bacteria^21–24^. It is an easily reproducible and widely used method for the investigation of the skin barrier^22,25–27^, and it allows for repeated or continuous measurements on the same patient without any discomfort or scarring, and thus represents a significant advantage to previously used methods (biopsies).

This is, to the best of our knowledge, the first time hBD-3 is detected in stratum corneum, using a minimally invasive technique^28^. hBD-3 has previously been shown^10,11,13,16,19^, in the skin barrier from skin biopsies, hBD-2 and LL-37 were previously detected using tape stripping^7,14,15^, and washing fluid^16^, and RNase7 and psoriasin were previously determined in skin washing fluid^16,29^.

Lesional AD skin showed great inter-individual variation in AMP concentrations, most likely due to inflammation and (local) differences in disease severity, hampering comparison between AD lesional skin and healthy skin. However, samples from non-lesional AD skin showed less inter-individual variation and exhibited high levels of hBD-3 and RNase7 as compared to healthy skin. This finding indicates that AD non-lesional skin is immunologically affected, despite lack of visible eczema lesions, and also implies that baseline levels of all AMPs are not downregulated in AD. Whether concentrations of AMPs in AD skin are sufficient for creating optimal antimicrobial defense is however still uncertain, as the concentration in normal, healthy skin when challenged by infections is yet to be determined. Colonization with *S. aureus* is frequent in AD-patients, affecting lesional as well as non-lesional skin^30^, and may partly explain the higher hBD-3 and RNase7 concentrations in AD skin, since hBD-3 and RNase7 have been shown *in vivo* to be of importance in relation to *S. aureus* infections^31,32^. In healthy skin, detectable levels of hBD-3 and RNase7 may possibly reflect a baseline expression of these AMPs, as part of the first line defense of the skin, and therefore constitutively expressed.

hBD-2 and psoriasin were predominantly detected in lesional skin, and only sporadically detected in AD non-lesional skin and healthy controls, confirming previous reports^10,16,17,29^. This finding indicates that these AMPs are more influenced by inflammation or skin barrier disruption, as seen in AD lesional skin, rather than a part of the constitutively expressed first line defense.

Many factors have proven to influence AMP function like pH^33^, protease activity^34^ and whether the peptides is in reduced or oxidized form^35^. Also, *in vitro* studies have shown keratinocytes isolated from AD patients to be insufficient in mobilizing stored hBD-3 from the cytoplasm of the cell to the surface of the bacteria^31^.

The use of repeated tape stripping of cell layers/corneocytes, for successively removal of the stratum corneum is widely used^22,25–27,36–38^, and TEWL is known to increase with increased number of tape stripping^26,27^, as also confirmed in our present study, illustrating a gradually induced impairment of the skin^39,40^. Previously, the removal of stratum corneum on the volar forearm by use of tape stripping has been reported to be accomplished after 20–40 tape strips and with the visual appearance of a shiny, red and dry surface^26,36,41,42^. The removal of stratum corneum is also confirmed by confocal microscopy after tape stripping^43^. Variation in stratum corneum removal is reported to be influenced by anatomical site, age, individual stratum corneum thickness and type of tape^44–46^. To minimize variation in sample collection, all samples in the present study were collected by the same investigator. Furthermore samples were collected from the same anatomical location (middle volar forearm), all participants were Caucasians, and sample collection standardized by tape (D-squame sample disc 22 mm) and pressure applied (225 g/cm^2^, CuDerm pressurizer). The use of tape stripping for collection of stratum corneum is widely used and accepted^7,14,21,47,48^, and we have used one of the most commonly used standardized tapes (D-squame^7,49–53^) together with standardized pressure to ensure reproducibility and validity of the method.

Distinguishing between different layers within stratum corneum should be interpreted with some caution, since cells on one tape strip may be derived from different layers of the skin due to skin furrows^54^ which are unavoidable. However, the findings indicate a relatively even distribution of AMPs in stratum corneum, and the differentiation of different layers in stratum corneum by tape strip samples have been reported previously^14^. In the present study advancement into the stratum corneum was apparent by the increase in TEWL after tape stripping. Two patients had only a minor increase in TEWL, which is attributed to individual differences in stratum corneum thickness, in agreement with previous reports^26,36^. Decreasing total protein levels, with increasing stratum corneum depth, as we have previously presented^24^, further indicates the advancement into depths of stratum corneum^26,36,55,56^. This is anticipated to be due to stronger cohesion between keratinocytes in the deeper layers of epidermis, thereby minimizing the amount of cells attached to the tape. The uniform concentrations of most AMPs in different depths indicate homogenous antimicrobial defense in stratum corneum. Future studies should include imaging methods to illustrate the precise depth/localization in the skin in relation to tapes removed.

Interestingly, we were not able to detect LL-37 in tape strips, which has been reported previously^7^. This discrepancy most likely reflects differences in extraction techniques between our methods, or it might be the detection limit of the commercial ELISA, as in a previous study successful in showing LL-37, an in-house immuno dot blot was used to detect LL-37 on tape strips (16). We also did not detect psoriasin as extensively as previously reported in a study using a washing fluid technique for AMP sampling and in-house ELISA^16,29^. However, in the present study the first tape strip was not included in the analyses which might explain these differences, since we detected psoriasin primarily in the outermost layers.

The finding of increased levels hBD-3 in AD non-lesional skin, compared to healthy skin, indicates that AD patients may not suffer from a general baseline deficiency in AMPs. The localization of several AMPs within different depths of stratum corneum, illustrates an evenly distribution of the innate immune defense throughout the skin barrier. Detailed knowledge about localization of different AMPs in the stratum corneum will contribute to improved understanding and insight in the role of AMPs in the naïve immune defense, and hence contribute to understanding the pathogenesis of inflammatory skin disease like AD. Further insight into the role of AMPs in inflammatory skin diseases may potentially harbor new targets for prevention and treatment of inflammatory skin diseases such as AD.

## Material and Methods

### Study population

Nine adult AD patients, six women and three men, age range 19–67 years, and five healthy controls, three women and two men, age range 21–59 years, were included in the study. The patients did not receive any systemic treatment for AD or any topical or systemic antibiotics for at least three months before enrollment in the study. All patients had used topical corticosteroid within 6–30 days from the sample-day, but paused for at least six days prior to sampling. Inclusion criteria were: > 18 years, AD according to UK criteria^57^, exclusion criteria were: breastfeeding or pregnant, and UV- therapy within 8 weeks. Healthy volunteers were without any history or manifestations of atopic disease or other skin diseases. The National Committee in Health Research Ethics, Copenhagen, Denmark, approved the study (H-1–2014–039), and the study was carried out in accordance with this approval. All participants provided informed written consent. Data on clinical parameters (SCORAD and TEWL) was previously presented in association with determination of proteins on tape strips^24^.

### Clinical characteristics of patients

Disease severity of AD was assessed using SCORAD (score range 0–103)^58^. Scoring is based on extent of eczema together with six clinical features: erythema, oedema/papulation, oozing/crust, excoriations, lichenification and dryness as well as two subjective symptoms: prurigo and sleep quality^59^. Skin barrier function was assessed by measurement of trans epidermal water loss (TEWL), evaluating the passive diffusion of water through stratum corneum. Measurements were taken on non-lesional skin, on the volar side of the middle forearm, using Dermlab open chamber Evaporimeter (Cortex Technology, Hadsund, Denmark), according to guidelines^60^. All measurements of TEWL were performed in triplicates and the registered values indicate the mean of the three measurements. Measurements were performed by the same investigator under standardized conditions, and patients were given time to adapt to room conditions before any measurements were carried out.

### Collection of stratum corneum using tape stripping

Collection of stratum corneum was standardized, using the same tape (D-squame®, CuDerm, Dallas TX, USA), pressed against the skin for 10 seconds using a standardized pressurizer of 225 g/cm^2^ (D500 - D-squame® pressure instrument, CuDerm, Dallas, TX, USA), and all collected by the same investigator from the same anatomical site^24^. All stratum corneum samples from AD non-lesional skin and healthy controls were collected from the middle of the volar forearm. Samples from AD lesional skin were collected from an eczematous skin area on the arm or leg. From each skin site, 36 consecutive tape strips were collected on the same spot. The first tape (tape 0) was not included in the analyses, since they were saved and stored for subsequent analyses. The following 35 tapes were pooled in groups of 5: tape 1–5 (depth 1), tape 6–10 (depth 2), tape 11–15 (depth 3), tape 16–20 (depth 4), tape 21–25 (depth 5), tape 26–30 (depth 6), and tape 31–35 (depth 7), hence 7 consecutive depths of stratum corneum were analyzed for each participant. The last tape visually had no skin remnants on it and visually the skin appeared shiny, red and dry indicating the removal of stratum corneum^22,38^.

Each tape was placed in tubes on ice, and PBS Buffer (1 mL) was added to samples, followed by 15 min of sonication in iced water (0–4 °C) in an ultrasonic bath (Bransonic® 5510, Branson Ultrasonics, Danbury, CT, USA). Samples were aliquoted into Eppendorf tubes and stored at −80 °C.

### Determination of total protein in stratum corneum samples

Protein determination was performed on tape strip samples of stratum corneum as previously described^24^, using a Micro BCA^TM^ Protein Assay Kit (Thermo Fisher Scientific Inc., Waltham, MA USA).

### Determination of AMPs in stratum corneum

Concentrations of AMPs in stratum corneum were determined using commercially available standard sandwich ELISA kits. Concentration of hBD-2 was analyzed with Human Beta Defensin 2 ELISA range 3.125–200 pg/ml (00–250-BD2, Alpha Diagnostic, San Antonio, USA). HBD-3 was determined using ELISA DEFb103A, standard range 46.88–3000 pg/ml, minimum detection limit 19 pg/ml (SEE132Hu, Cloud Clone Corp, USCN, USA). RNase7 concentration was analyzed with ELISA RNASE7, standard range 1.55–100 ng/ml, minimum detection limit 0.55 ng/ml (SED193Hu, Cloud Clone Corp, USCN, USA). Psoriasin with S100A7 ELISA, standard range 125–8000 pg/ml, minimum detection limit 50 pg/ml (SEC. 03Hu, Cloud Clone Corp, USCN, USA), and LL-37 with ELISA CAMP, standard range 1.23–100 ng/ml, minimum detection limit 0.55 ng/ml (CEC149Hu, Cloud Clone Corp, USCN, USA). To standardize concentrations of AMPs, these are calculated as AMP/µg of total protein in each sample.

### Validation of commercial ELISA - Spike tests

ELISAs were validated by testing recombinant synthetic peptide for each ELISA, which all gave positive results. For hBD-3, CAMP and psoriasin a Quality Control was obtained, made from human serum containing human AMP in known concentration. The following spike tests were applied: For hBD-2 ELISA: 1) Recombinant hBD-2 (RPA072Hu01, Cloud Clone) and 2) Synthesized hBD-2 (HBD24-P, Alpha Diagnostic). For ELISA hBD-3: 1) Quality Control (Cloud Clone) containing human DEFb103A, 2) Recombinant DEFb103A (RPE132Hu01 Cloud Clone) and 3) synthesized HBD-3 (HBD38-R-25, Alpha Diagnostics). For ELISA RNase7: 1) Recombinant RNase7 (RPD193Hu01, Cloud Clone), and 2) recombinant peptide added to tape. For ELISA CAMP: 1) Quality Control (Cloud Clone) containing human CAMP, and 2) Recombinant peptide (RPC419Hu01, Cloud Clone).

### Statistical analyses for AMPs

Mann-Whitney non-parametric test was used to detect differences between AD skin and healthy control skin, Wilcoxon matched-pairs test was used to detect differences between lesional and non-lesional skin in AD patients. Statistical analyses were carried out using Prism 6 Graph Pad.

### Data availability

The datasets generated during and/or analyzed during the current study are available from the corresponding author on request.

### What is already known

- The amount of certain AMPs is decreased in atopic dermatitis skin relative to psoriasis
- Atopic dermatitis patients suffer from a compromised skin barrier

### What does this study add

- Using a minimally invasive technique, measurements of AMPs in stratum corneum are performed.
- AMPs are assessed in consecutive depths of the outermost skin layers.

## Electronic supplementary material

Supplementary Dataset
