# Supplementary material for: Measurements of AMPs in stratum corneum of atopic dermatitis and healthy skin–tape stripping technique
Source: Sci Rep. 2018 Jan 26;8:1666. doi: 10.1038/s41598-018-20204-8 (PMC5786105; doi:10.1038/s41598-018-20204-8)
Supplement: Supplementary file 1 — Supplementary Dataset [file 41598_2018_20204_MOESM1_ESM.doc]

**Supplementary data:**

**Measurements of AMPs in stratum corneum of atopic dermatitis and healthy skin – tape stripping technique**

Maja-Lisa Clausen*^1^, H-C Slotved^2^, Karen A. Krogfelt^2^, Tove Agner^1^

**Supplementary table S1: Concentration of AMPs at each stratum corneum depth**

Median concentration of each antimicrobial peptide (AMP) at every stratum corneum depth, for atopic dermatitis lesional skin (AD ls) and non-lesional skin (AD nls) as well as healthy control (HC) skin. For hBD-2 and hBD-3 stable levels through the depth of stratum corneum is observed, but for RNase7, a tendency to increasing levels with increasing depth is seen in AD lesional skin. Results are given as median values of 9 AD samples and 5 HC samples for each stratum corneum depth.

| Stratum Corneum | hBD-2  *pg/µg protein* | | | hBD-3  *pg/µg protein* | | | RNase7  *ng/µg protein* | | | Psoriasin  *pg/µg protein* | | | LL-37  *ng/µg protein* | | |
| --- | --- | --- | --- | --- | --- | --- | --- | --- | --- | --- | --- | --- | --- | --- | --- |
|  | AD ls | AD nls | HC | AD ls | AD nls | HC | AD ls | AD nls | HC | AD ls | AD nls | HC | AD ls | AD nls | HC |
| Depth 1 | 0.97 | 0 | 0.21 | 158.5 | 270.1 | 97.9 | 2.0 | 3.7 | 2.1 | 2.1 | 0 | 0 | nd | nd | nd |
| Depth 2 | 2.14 | 0 | 0 | 235.6 | 192.3 | 67.6 | 2.4 | 2.5 | 2.0 | 0 | 0 | 0 | nd | nd | nd |
| Depth 3 | 1.63 | 0 | 0 | 331 | 214.7 | 152.4 | 2.7 | 2.9 | 2.1 | 0 | nd | 0 | nd | nd | nd |
| Depth 4 | 0.68 | 0 | 0 | 565 | 358.4 | 183.6 | 3.7 | 4.6 | 2.3 | 0 | nd | 0 | nd | nd | nd |
| Depth 5 | 0 | 0 | 0 | 294.2 | 204.9 | 165.2 | 3.6 | 2.9 | 1.8 | 0 | nd | nd | nd | nd | nd |
| Depth 6 | 1.04 | 0 | 0 | 359.4 | 241.5 | 110.5 | 5.5 | 2.6 | 2.3 | 0 | nd | nd | nd | nd | nd |
| Depth 7 | 1.49 | 0 | 0 | 335.5 | 196.7 | 100.6 | 5.4 | 2.2 | 2.3 | nd | nd | nd | nd | nd | nd |

nd – not detected

**Supplementary figure S2:** Spike test for hBD-2, hBD-3, Psoriasin, RNase7 and LL-37.

CC = Cloud Clone; Alpha = Alpha Diagnostic; QC = Quality Control;
